# International Prevalence and Correlates of Psychological Stress during the Global COVID-19 Pandemic

**DOI:** 10.3390/ijerph17249248

**Published:** 2020-12-10

**Authors:** Maheen M. Adamson, Angela Phillips, Srija Seenivasan, Julian Martinez, Harlene Grewal, Xiaojian Kang, John Coetzee, Ines Luttenbacher, Ashley Jester, Odette A. Harris, David Spiegel

**Affiliations:** 1 Rehabilitation Service, Veterans Affairs Palo Alto Health Care System, Palo Alto, CA 94304, USA; aphill73@stanford.edu (A.P.); srija.seenivasan@va.gov (S.S.); martinez.julian.sf@gmail.com (J.M.); Harlene.Grewal@va.gov (H.G.); xkang@pavir.org (X.K.); Jpcoetzee@stanford.edu (J.C.); 2 Department of Neurosurgery, Stanford School of Medicine, Stanford, CA 94305, USA; Odette@stanford.edu; 3 Department of Psychiatry & Behavioral Sciences, Stanford School of Medicine, Stanford, CA 94305, USA; dspiegel@stanford.edu; 4 Department of Psychology, University of Amsterdam, 1001 NH Amsterdam, The Netherlands; Ines.Luttenbacher@gmail.com; 5 Science and Engineering Libraries, Stanford Libraries, Stanford University, Stanford, CA 94305, USA; ajester@stanford.edu

**Keywords:** COVID-19, coronavirus, global, psychological stress, mental health

## Abstract

This study reports perceived stress and associated sociodemographic factors from an international sample of adults, during the COVID-19 pandemic. The Perceived Stress Scale (PSS-10) along with socio-demographic questions were conducted between 8 April 2020 and 11 May 2020. The survey was translated from English into five languages. Recruitment was conducted worldwide using social media. A total of 1685 survey responses were collected across 57 countries with eleven countries (≥30 responses/country) included in the sub-analyses. Overall, the mean PSS-10 score was 19.08 (SD = 7.17), reflecting moderate stress compared to previously reported norms. Female gender was associated with a higher PSS score (3.03, *p* < 0.05) as well as four-year degree holders (3.29, *p* < 0.05), while adults over 75 years (−7.46, *p* < 0.05) had lower PSS scores. Personal care composite score (including hours of sleep, exercise, and meditation) was associated with lower PSS scores (−0.39, *p* < 0.01). Increases in personal care and changes in work expectations were associated with lower PSS scores (−1.30 (*p* < 0.05) and −0.38 (*p* < 0.01), respectively). Lower total PSS scores were reported in Germany (−4.82, *p* < 0.01) compared to the global response sample mean. This information, collected during the initial period of global mitigation orders, provides insight into potential mental health risks and protective factors during crises.

## 1. Introduction 

### 1.1. Background

Following the COVID-19 outbreak, the world has experienced an international crisis that is unprecedented in recent history, resulting in many forms of regional lockdowns or social restrictions. Governments worldwide have adopted a series of mitigation procedures (e.g., quarantine, social distancing, and isolation) impacting over half of the world’s population [1]. Previous research during and after other pandemics, natural disasters, wide-scale trauma, and acts of terrorism has measured the impacts of isolative measures on mental health, worldwide [2,3,4,5]. Reports have also shown associations between poorer mental health outcomes and quarantine length, which can persist over several years [2,3,4,5,6]. The importance of early intervention, identification of risk and protective factors contributing towards the development of mental health challenges (e.g., post-traumatic stress), and how individuals adapt when exposed to large-scale traumatic events (i.e., resilience) all aid in understanding the widespread psychological impacts of such events while informing potential interventions addressing these needs [2,3,4,5,6]. As prior work assessing the effects of acute disasters has commonly measured outcomes of depression and post-traumatic stress disorder (PTSD), the COVID-19 pandemic has continued to impose an ebb and flow of both traumatic and non-traumatic life changes, giving rise to potentially unique sets of psychosocial stressors that may contribute towards individual, psychological outcomes yet to be seen [7,8]. 

Although limited in number, recent reports have addressed the psychological impacts of COVID-19, including “post-traumatic stress symptoms, confusion, and anger” caused by the length of quarantine isolation, fear of infection and mortality, boredom, lack of supplies, inadequate information, financial loss and/or stigma [4,6,9,10,11,12,13,14,15]. The psychological impact of COVID-19 has been rated as moderate or severe by 53.8% of the population in Wuhan, China [6], with similar reports emerging from Italy, and the United States (US) [9]. Additionally, US preliminary data show that “shelter in place” orders were associated with greater health anxiety, loneliness, and financial worry [12,15]. Limcacoco and colleagues [12] conducted a global survey during the earlier stages of the pandemic (17 March to April 2020) and also predicted higher stress levels due to the pandemic. Despite different scales used in these studies (e.g., Perceived Stress Scale (PSS-10); Depression Anxiety Stress Scales (DASS-21)), the COVID-19 pandemic highlighted several demographic and risk and protective factors that are important for the management of the current mental health crisis.

For instance, international samples have consistently associated higher perceived stress with being female [11,12,15,16,17] and a young adult, while older adults (>60 years) show more mixed results [12,16,17]. Factors mediating stress have included remote work status, knowing someone with COVID-19, and income level [6,9,11,15,18]. Data supporting a potentially significant increase in working from home (WFH) have also been reported worldwide since the pandemic outbreak, with the ability for approximately 29–34% of individuals to fully WFH in countries such as Argentina, Germany, Sweden, UK and the US [19,20]. As women already tend to be primary childcare providers, it is of particular interest as to how the potential increase in working from home, homeschooling and number of dependents will impact their perceived stress, compared to men [21].

Despite the many pandemic-related challenges that may occur, coping strategies and positive events are important to identify. For example, finding access to emotional and social support has been associated with positive outcomes in prior disaster- and trauma-related research such as the H1NI flu pandemic in Canada [17] and post-traumatic outcomes after the Severe Acute Respiratory Syndrome (SARS) epidemic in Singapore [22]. Similarly, coping strategies including social and emotional support predicted six-month outcomes in a US-based survey after the 11 September 2001 terrorist attacks [5]. In light of more significant mental health concerns, continued phases of mitigation procedures and lack of a COVID-19 vaccine, it is essential to establish the prevalence of stress and identify the negative and positive coping factors world-wide, in relation to socio-demographic factors. Of additional importance to potential coping strategies during the COVID-19 pandemic may be daily routine and exercise, which have shown to boost immune response and overall well-being [23]. It is less clear as to how other factors such as meditation and behavioral activation may serve as coping strategies during stressful and isolative periods [24]. With so many potential impacts resulting from the COVID-19 pandemic, it is essential to identify stress levels along with risk factors and coping strategies to address the rising mental health concerns worldwide.

### 1.2. Rationale and Hypotheses

**Figure 1:**
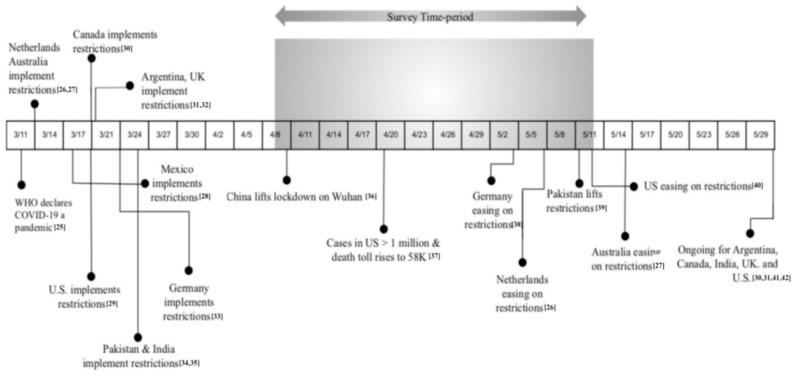
Survey timeline in the context of relevant COVID-19 pandemic events in 2020 [25,26,27,28,29,30,31,32,33,34,35,36,37,38,39,40,41,42].

Our primary objective was to capture the level of perceived stress in relation to sociodemographic factors and changes in daily life during the worldwide COVID-19 pandemic (see Figure 1). Our secondary objective was to test the relationship between perceived stress and measures of personal care (e.g., exercise, meditation, sleep and time spent with family and friends through telecommunication) and personal burden (e.g., remote work, homeschooling and number of dependents) reported by survey participants. Based on our literature review, such personal care factors have been suggested as protective, while the identified personal burden questions may pose greater risk for increased stress; however, these factors have yet to be investigated during this pandemic. We predicted that worldwide perceived stress would be significantly higher compared to norms reported prior to the current pandemic. Based on prior literature, we also predicted higher average stress in females compared to males, and lower average stress associated with personal care factors.

## 2. Materials and Methods

### 2.1. Study Design and Respondents 

The study was conducted in accordance with the Declaration of Helsinki. All adults over 18 gave their informed consent for inclusion before they participated in the study. A cross-sectional 38-item, 8–10 min electronic survey (see Supplementary Materials) made up of demographic, personal care, personal burden questions, and the Perceived Stress Scale (PSS-10), was approved by the Stanford Institutional Review Board (IRB) and delivered through the Qualtrics^®^ (Provo, UT, USA) online survey platform [43]. Survey data were collected anonymously from 8 April to 11 May 2020 (33 days), with no monetary incentive given. A survey completion bar and midpoint text stating “You are halfway” were added to retain the participants’ attention. The Qualtrics^®^ platform distributed the survey worldwide across all translations after the initial launch of the survey in English. The survey was distributed globally using a snowballing convenience sampling technique via social media, university networks and personal contacts. We first posted on our laboratory website and initially advertised this link worldwide through Facebook^®^ (Menlo Park, USA), LinkedIn^®^ (Sunnyvale, USA), WhatsApp^®^ (Mountainview, USA) emails and university networks in the US, UK, Netherlands, China, India, Pakistan, and Mexico (note: we did not select a country beforehand and included all countries who responded). We then chose six other languages for translation based on the countries we had no response from after our initial launch. The PSS-10 is adapted from the original 14-item PSS and has been validated and translated in several languages, with population norms across languages and cultures [43]. We used the existing translation for PSS-10 in the following six languages: Spanish, traditional and simplified Chinese, French, Italian and Japanese. We then used Google^®^ translate for the remaining pandemic-related survey questions. These additional translations were then confirmed and edited by native speakers in each language chosen from an academic setting (see Supplementary Table S1). Participants were eligible if they read and agreed to the consent form and confirmed their age (≥18 years). European Union (EU) respondents were also informed of their data protection rights (see Supplementary Materials). Participants were required to respond to all items on the first three pages to move forward with the survey. 

**Figure 2:**
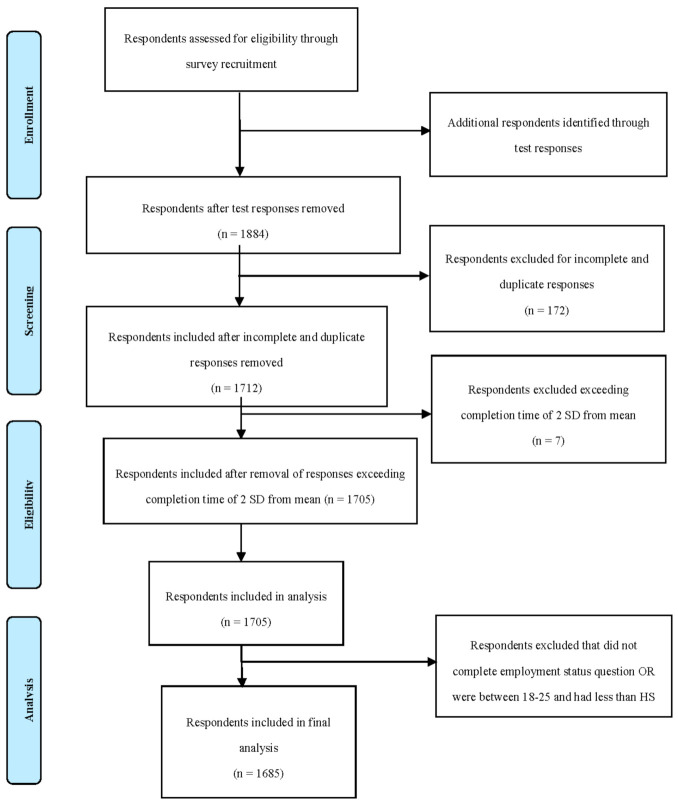
Participant flow diagram.

A total of 1884 surveys were completed between 8 April and 11 May 2020, with a final sample of 1685 after meeting inclusion/exclusion criteria (Figure 2). The median time to complete the survey was 320 s (5 min, 20 s); the mean time was ~579 s (9 min, 39 s). As shown in Figure 2, responses were eliminated if they were: pilot testing by lab members, incomplete or duplicate, exceeding completion time of 2 SD away from the mean based on Woods et al., 2017 [44]; and did not complete employment status question OR were between 18–25 and had less than high school (based on reviewer’s suggestions). Overall, most of the respondents were female (*N* = 1047) and the average age range of the largest number of respondents (25.46%) was 25–34 years old. Effect size calculation was used to determine a minimum sample size for the global response sample [45]. A minimum sample size of 116 was shown to be sufficient in order to achieve a significance level α = 0.05 and a medium effect size of 0.35 in a multiple regression analysis with 25 predictors, 0.95% power, and fixed effects.

### 2.2. Measures 

The 38-item online survey included socio-demographic, personal care and personal burden questions, and the PSS-10 English version (see Supplementary Materials) All survey versions in different languages included the same questions. Survey items 1–12 included socio-demographic items such as marital status, income (in each country’s currency), age, and education. Gender was specified rather than sex to report the psychosocial factor rather than the biological factor. Survey items 13–20 included personal care and personal burden questions: homeschooling, dependent care, remote work, sleep, exercise, and meditation. Survey items 21–27 asked whether the following had changed due to COVID-19: work expectations, remote work, sleep, meditation, exercise and telecommunication with friends and family. The final survey items, 28–38, consisted of the PSS-10—a widely used tool that measures the perception of stress in response to unpredictability, overload, and lack of control [43]. Each question asks about the frequency of feelings and thoughts related to each item during the last month and is rated on a 5 point Likert-scale ranging from 0 (never) to 4 (very often). Questions 4, 5, 7, and 8 are reverse scored. The PSS-10 total score is then categorized as low stress (0–13), moderate stress (14–26), or high stress (27–40). 

### 2.3. Statistical Analysis

Statistical analyses were performed using statistical program STATA (StataCorp, College Station, TX, USA). To identify the level of perceived stress worldwide in relation to sociodemographic factors and changes in daily life in response to the COVID-19 pandemic, a threshold of 30 responses per country was required for analysis inclusion [45]. Survey responses were received from 57 countries, with 11 countries meeting the inclusion criteria for further analyses. For the US data, we created four census sub-regions: northeast, midwest, west, and south. Data are reported based on results from these four sub-regions.

Descriptive data for primary variables of interest such as demographics, daily remote work hours, healthcare industry workers, homeschooling and dependents are presented in Table 1. Multiple ANOVAs were conducted on descriptive data to investigate if any significant differences existed in sociodemographic data worldwide. The overall fit is an F-statistic (which applies to the pairwise Analysis of Variance (ANOVA) and the Multivariate Analysis of Variance (MANOVA) as models), but the comparisons reported between groups (mean vs. mean) are t-statistics.

A factor analysis on all PSS-10 items confirmed a one-factor model as shown in previous literature; therefore, the PSS-10 total was used as the primary outcome. Composite variables were constructed for personal care; personal burden and increase in personal care compared to before COVID-19. Specifically, personal_burden = (homeschool_covid + homecare_covid + remote_covid); personal_care = (sleep_covid + exercise_covid + meditation_covid + fam_friends_connect_covid); and, Increase_in_personal_care_composite = (more_less_sleep + more_less_exercise + more_less_meditation + more_less_fam_friends_connect)/4.

The PSS-10 total was used as an outcome in a Tobit regression model with demographics, composites for personal care, personal burden and increase in personal care due to COVID-19 as covariates. We also included knowing someone with COVID-19, work expectations changed due to COVID-19, and daily remote work hours in the model. Countries were represented as dummy variables, though not all dummies could be included due to collinearity with other covariates. The level of significance was set at *p* < 0.05, and explicitly provided when the value was *p* < 0.01 and *p* < 0.001. Confidence intervals are also reported. The race/ethnicity questions are only applicable to the US. For this very reason, we did not use racial/ethnicity questions in other countries and did not include them in our international analysis with US regions included.

## 3. Results

**Figure 3:**
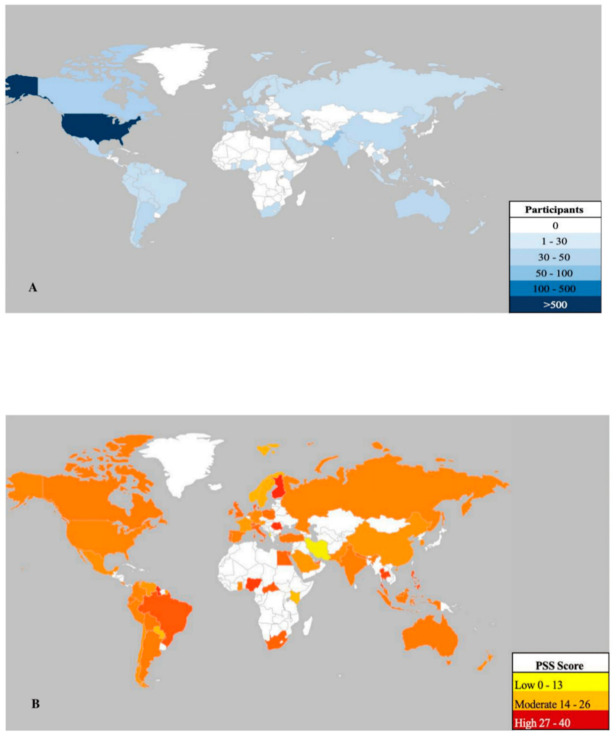
Worldwide representation of perceived stress levels as measured by the Perceived Stress Scale (PSS-10). (**A**) represents number of respondents in the survey in countries including: Albania, Argentina, Australia, Austria, Bolivia, Brazil, Canada, Central African Republic, Chile, China, Colombia, Costa Rica, Denmark, Ecuador, Egypt, Finland, France, Germany, Ghana, Guyana, India, Indonesia, Iran, Ireland, Israel, Italy, Kenya, Luxembourg, Malaysia, Mexico, Nepal, Netherlands, New Zealand, Nigeria, Norway, Pakistan, Paraguay, Peru, Philippines, Poland, Portugal, Romania, Russia, Saudi Arabia, Singapore, South Africa, South Korea, Spain, Sweden, Switzerland, Thailand, the former Yugoslav Republic of Macedonia, Turkey, United Arab Emirates, United Kingdom, United States of America, Venezuela. (**B**) represents the mean PSS-10 total score in each of the countries shown in panel A. Note: the map was constructed using the Geographical Heat Map application, publisher Keyur Patel.

Overall: 

A total of 1685 survey responses were collected across 57 countries (Figure 3A—heat map) including: US (869/51.20%), Pakistan (153/8.97%), Canada (88/5.16%), Netherlands (81/4.75%), Germany (53/3.17%), Argentina (44/2.64%), Mexico (47/3.05%), Australia (45/2.76%), UK (38/2.23%), India (38/2.23%), China (33/1.99%) and all others (196/11.63%) (see Table 1). The overall mean PSS-10 score worldwide was 19.08 (SD = 7.17) (moderate stress level) (Figure 3B—heat map). Overall, females responded more than men and “other” gender (1047/62.14%) and a greater number of people reported working remotely than not (1141/67.72%). The PSS-10 total mean score was significantly different between: gender categories (F = 35.87 *p* < 0.01), age categories (F = 20.93, *p* < 0.001), employment status (F = 10.08, *p* < 0.01), and income brackets (F = 2.38, *p* < 0.05). Stress did not differ significantly by education, industry, or number of dependents (*p* > 0.05). Total respondents per country and mean PSS scores per country are shown in Table 1.

The Tobit regression (Table 2) was applied to the PSS-10 total score as the dependent variable while the reference categories (i.e., male, 18–24, with high school diploma, fourth quartile income, employed, with zero dependents) were added as covariates. The overall model accounted for variance in perceived stress levels (R2 = 0.04). Note that although the results are very robust statistically, our observed covariates only explain about 4% of observed variance by the R-squared measure. Adults over 75 years showed lower perceived stress (b = −7.13 *p* = 0.05, [95% CI −14.29 to −0.03]), females showed more stress than males (b = 3.17, *p* < 0.000 [95% CI 2.19 to 4.16]), as did some college/no degree (b = 3.25, *p* < 0.05, [95% CI 0.02 to 6.48]) and four-year degree holders (b = 3.28, *p* < 0.05, [95% CI 0.18 to 6.38]); doctoral degree holders showed a trend for more stress (b = 2.74, *p* < 0.01, [95% CI −0.44 to 5.92]) as did students (b = 1.82, *p* < 0.01, [95% CI −0.15 to 3.79]). Furthermore, also associated with lower perceived stress was the personal care composite (b = −0.39, *p* < 0.00, [95% CI −0.50 to −0.28]), increase in personal care since COVID-19 composite (b = −1.33, *p* < 0.05, [95% CI −2.35 to −0.30] and changes in work expectations due to COVID-19 (b = −0.40, *p* < 0.01, [95% CI −0.67 to −0.12]). 

Marital status, employment status, income bracket, personal burden composite, daily remote work hours, and knowing someone with COVID-19 did not influence stress. In comparison to the global response sample average, Germany showed lower stress (b = −4.63 *p* < 0.01, [95% CI −7.68 to −1.58]), followed by Pakistan (b = −2.43 *p* < 0.05, [95% CI −4.58 to −0.28]) and then Mexico (b = −2.75 *p* < 0.05, [95% CI −5.48 to −0.03]). Additionally, none of the four US census sub-regions showed a significant difference in stress levels compared to the global average (west region showed a trend (*p* > 0.05).

## 4. Discussion 

The current study aimed to capture the level of perceived stress in relation to sociodemographic factors and changes in daily life due to the COVID-19 pandemic. Our results echo recent reports that have highlighted the rise in perceived stress across the world [4,10,46]. Our overall mean score (19.08, SD = 7.17) was higher than what was preliminarily reported in Limcaoco et al., 2020 (17.40, SD = 6.40)—a global survey that ended on 1 April 2020. Reported PSS-10 norms prior to the pandemic showed an average score of 12.89 in Germany, 15.81 in Mexico, 19.25 in India, 19.2 in China, 19.79 in UK, and 15.05 in the US [46,47,48,49,50,51]. Compared to normative PSS-10 data, our means showed an overall trend of higher averages in the country-level analyses we report (e.g., Germany and Mexico; we were unable to find pre-pandemic PSS-10 data for Pakistan) [12,47,52,53]. This may be due to the specific time window and duration (i.e., 33 days) of our survey compared to surveys conducted previously. For instance, one was conducted earlier in the pandemic, while others were of a shorter duration [6,9,11,12,16]. Our PSS-10 responses suggest highly prevalent feelings of uncertainty, stress, anxiety, and lack of control over one’s life in response to the pandemic. We believe that our data, collected over 33 days during the largest pandemic in history, document a shared experience of perceived stress in international communities.

### 4.1. Sociodemographic Factors and Perceived Stress

#### 4.1.1. Gender and Age

Consistent with prior PSS-10 studies, females report greater perceived stress than men worldwide [12,13]. This has been observed in previous epidemics and in other recent COVID-19 studies [6,9,11,12,13,16]. Adults over 75 years of age report lower stress despite being at higher risk for the worst outcomes from COVID-19, similar to other recent studies (although different from findings in China) [6,9,11,12,13,16,54]. Additionally, our study identified females (worldwide) and younger adults (within the US) as being at higher risk for perceived stress [10].

#### 4.1.2. Education

Internationally, some college/no degree and four-year degree holders showed higher perceived stress compared to high-school degree holders. Previous studies have reported mixed results for the effect of education on stress across the globe. For instance, education did not influence mental health among two recent Chinese survey studies [13,55]. However, an association between education and stress was found in a Spanish sample during the outbreak [14].

#### 4.1.3. Race, Ethnicity, and Income

The survey data collected worldwide could not specifically address race disparities within each country and did not allow for a reliable analysis of race or ethnicity, and therefore we decided not to report the results. Similarly, the lack of correlation between income and stress may be a result of our collection strategy, which yielded more respondents from middle- and high-income level groups. Although lower income has been associated with increased psychological stress, such individuals in some countries received substantial government aid during the pandemic, as is the case in Germany, which could offset any association of lower income with perceived stress [10,56].

#### 4.1.4. Remote Work

Changes in remote work schedules lowered stress levels within the US and worldwide, with over a third of employed US respondents turning to remote work [57]. In Germany, remote work was more commonplace prior to the pandemic, making the large-scale transition to such work easier than it may have been in other countries [56]. As previous reports projected up to 34% of individuals being able to WFH, our 44% remote work sample may reflect the greater and temporary increase during heightened mitigation procedures and not individuals who will have fully remote roles [20]. However, it is important to consider the potential of a continued shift towards this remote model in a post-COVID economy. Additionally, our time period captured a 33 day period during which people may have initially started remote work schedules, feeling relief in reduced exposure to the virus.

### 4.2. Personal Care/Burden and Perceived Stress

Our secondary goal was to explore the relationship between personal care and personal burden factors that may be useful in mitigating perceived stress during the COVID-19 pandemic. In addition to the change in work situations, protective factors such as hours of sleep, meditation, and exercise were associated with less stress worldwide, and in the US. Consistent with recent studies, marital status, dependents, and knowing someone with the virus did not affect stress levels [6]. Other studies have reported that social support is an important factor contributing to perceived stress that is related to loneliness [18].

### 4.3. Limitations

Our primary limitation was recruitment strategy, as convenience-sampling was utilized via a snowballing strategy. This convenience sample is overweighted with individuals who have greater years of education, middle-to-higher-income earners, and who identify as white, which does not reflect a regional sample fully representative of general populations. Although our sample is statistically robust because of its size, we cannot make a population-based estimate from our results. This is primarily because we do not have sub-national information on any country, and we cannot perform a cluster analysis for aggregation. For this reason, we treated countries as dummy variables and not random effects. Our race and ethnicity questions are primarily applicable to the US. Therefore, we did not use racial and ethnicity questions in other countries and did not include them in our international analysis. Although we included the latest WFH numbers for the US and other countries to report on how many people were working from home during the time this survey was conducted, we assert that this does not provide a complete picture of WFH numbers across the world. We are not estimating the state of stress worldwide but we believe that our data do add to the existing literature on mental state and stress levels of those individuals across the world who were WFH during the early period of the pandemic.

Financial incentives were not offered to complete the survey; however, the survey length was limited to 8–10 min to reduce attrition and fatigue. Data were collected over a period of 33 days during the continuing pandemic, though mitigation procedures may have differed across countries throughout this period, influencing survey results. Substantial variation amongst countries with regard to additional resources (economic and otherwise) offered to citizens during lockdown may have impacted stress variability and was not included in our analysis. The PSS-10 items were given at the end of the survey as we did not want to bias the behavioral patterns adopted by respondents during this time, which may influence their response to stress questions. We also did not ask personal COVID-19-related questions to influence the response and asked an equal number of personal burden and care questions. Finally, we cannot speak to the pre-existing mental health challenges that survey respondents may have experienced, as this information was not collected.

## 5. Conclusions

Our primary goal was to capture levels of perceived stress across the globe in the timeframe of COVID-19 mitigation procedures where factors such as social isolation and sudden daily life changes could have created heightened perceived stress [4,10]. We believe that these data provide insight into potential mental health risks, as well as protective factors that may inform future efforts to manage mental health during crises. As prior studies have reported long-term, post-pandemic behavioral and psychological outcomes, meso and macro level changes may need to occur in order to address these ongoing challenges during and after the pandemic [6,10]. Additionally, mental health challenges may result from COVID-19 experiences such as moral distress and moral injury, caused by the profound practical and ethical challenges many individuals must confront during the pandemic, as well as the loss of trust in leadership and in larger systems that individuals adhere to and derive identity from [58].

Similar to previous studies, the oldest age group (>75) of our worldwide sample exhibited less perceived stress, which is likely a consequence of the better emotional regulation that previous research has found to be present in older adults [48]. Additionally, we identified being female as being associated with higher perceived stress [48]. We have also identified that behavioral factors such as sleep, meditation, and exercise appear to confer resiliency against greater perceived stress. Finally, living in specific countries (e.g., Germany, Pakistan, and Mexico) was associated with lower stress, and the cultural and policy differences that distinguish those countries should be examined. Collectively, these findings may contribute towards attempts to address mental health and personal burden and protective factors during future crises.

Although individuals in countries across the world have collectively experienced significant involuntary lifestyle changes and a concomitant rise in mental health problems, recent events have also resulted, in some cases, in a strengthening of government healthcare systems [10,16,18,47]. Medical provider and staff training in technology, virtual counseling and support is already underway. Medical innovations are currently experiencing a wider adoption into the healthcare system including at-home device usage, virtual reality, and telemedicine. Protective factors that improve self-efficacy and emotional stability may lead to better health outcomes during and after pandemic mitigation procedures. Future goals for researchers must include confirmatory analyses of findings such as those reported here by combining data from multiple surveys and including the influence of specific factors such as financial impact, social isolation, and access to healthcare, especially for disadvantaged minorities and at-risk populations. Once confirmed, efforts can be directed to these at-risk individuals for increased perceived stress such as offering more personal care services through mobile monitoring apps. This would allow follow-up and remote monitoring capabilities for at-risk populations, resulting in better mental health overall. Recovery from the 2020 global COVID-19 pandemic requires an effective and synergistic public health effort to monitor mental health across the world, which can also serve to help address future pandemics.

## Figures and Tables

**Table 1:** Demographics for Global Perceived Stress Scale (PSS-10).

Demographics	N	%	Mean	SD
**Total**	1685	100%	19.08	7.17
**Age (Years)**				
18–24 years old	279	16.56%	20.14	7.26
25–34 years old	429	25.46%	20.57	7.07
35–44 years old	381	22.61%	19.86	6.75
45–54 years old	310	18.40%	18.1	6.53
55–64 years old	173	10.27%	16.8	7
65–74 years old	77	4.57%	16.26	7.49
75 years or older	36	2.14%	10.36	5.64
**Gender**				
Male	627	37.21%	17.2	7.05
Female	1047	62.14%	20.17	7
Other	11	0.65%	21.91	7.84
**Marital Status**				
Married	841	49.91%	18.42	7.07
Widowed	20	1.19%	17.05	9.08
Divorced	88	5.22%	18.55	6.8
Separated	22	1.31%	18.59	6.7
Partnered	198	11.75%	20.2	7.61
Single	441	4.45%	19.92	7.13
Other	75	26.17%	19.91	6.29
**Education (Years)**				
High school graduate (high school diploma or equivalent including General Education Development (GED)	79	4.69%	19.24	8.76
Some college but no degree	223	13.23%	19.7	7.48
Associate degree in college (2-year)	83	4.93%	19.52	6.65
Bachelor’s degree in college (4-year)	526	31.22%	19.54	7.09
Master’s degree	425	25.22%	18.77	6.72
Doctoral degree	255	15.13%	18.1	7.01
Professional degree (Juris Doctor (JD), Medical Doctor (MD)	94	5.58%	18.54	7.88
Employment Status *				
Working (full-time)	977	55.07%	18.77	6.94
Working (part-time)	163	9.19%	19.61	7.21
Unemployed	104	5.86%	20.72	7.39
Retired	70	5.81%	22.2	7.47
Not working due to disability	103	1.30%	14.51	7.15
Student	23	13.59%	22.04	6
Other	241	5.24%	20.52	7.17
Laid off or looking for work due to COVID-19	93	3.95%	19.54	7.46
Countries *				
United States of America	869	51.20%	18.99	7.42
Pakistan	153	8.97%	18.27	6.39
Canada	88	5.16%	19.9	7.06
Netherlands	81	4.75%	18.94	7.87
Germany	53	3.17%	18.25	6.27
Mexico	47	3.05%	17.87	6.19
Australia	45	2.76%	20.2	7.08
Argentina	44	2.64%	19.86	7.27
United Kingdom	38	2.23%	21.32	6.51
India	38	2.23%	19.55	6.31
China	33	1.99%	17.15	4.47
Other Countries **				
**Total**	196	11.63%	19.70	7.20
**U.S. Regions**				
Northeast	99	12.4%	20.42	6.74
South	201	25.3%	19.39	7.6
Midwest	74	9.32%	18.98	7.96
West	420	52.90%	18.49	7.35
**Dependents**				
0	826	49.02%	19.32	7.48
1	242	14.36%	18.62	7.19
2	290	17.21%	19.33	6.82
3	166	9.74%	18.63	6.78
4	75	4.40%	19.04	6.64
5+	86	5.04%	18.09	6.28
**Remote Work**				
N/A	544	32.28%	18.88	7.61
<1 h	27	2.68%	17.59	6.42
1–5 h	241	23.91%	19.51	6.52
5–8 h	447	44.35%	18.98	6.75
>8 h	293	29.07%	19.14	7.24
**Homeschooling**				
Yes	517	30.68%	19.46	7.61
No	813	48.25%	18.64	7.31
N/A	355	21.07%	19.5	6.59
**Healthcare Industry Workers**				
Yes	180	10.68%	18.78	6.79
No	1505	89.32%	19.12	7.22

**Note.** * “Other Countries” included (countries with <30 responses): South Africa, Spain, Italy, France, Venezuela, Saudi Arabia, Brazil, Colombia, United Arab Emirates, Bolivia, Ecuador, Nigeria, Peru, Switzerland, Austria, Chile, Turkey, Costa Rica, Guyana, Norway, Paraguay, Romania, Russia, Singapore, Sweden, Albania, Central African Republic, Denmark, Egypt, Finland, Ghana, Indonesia, Iran, Ireland, Israel, Kenya, Luxembourg, Malaysia, Nepal, New Zealand, Philippines, Poland, Portugal, South Korea, Thailand, the former Yugoslav Republic of Macedonia. ** Respondents excluded that did not complete employment status question OR were between 18–25 and had less than high school education * (*n* = 20).

**Table 2:** Worldwide associations between sociodemographic variables and perceived stress during the COVID-19 pandemic.

Demographics	Beta Coef	Std Error	95% CI		*p*-Value
**Marital Status**					
Widowed	−2.75	3.89	−10.41	4.90	0.48
Divorced	−0.67	1.03	−2.08	1.95	0.95
Separated	−1.46	1.87	−5.15	2.22	0.43
Partnered	0.46	0.82	−1.15	2.06	0.58
Other	0.29	1.43	−2.51	3.10	0.21
Single	0.41	0.72	−1.01	1.82	0.56
**Age**					
25–34	0.85	1.00	−1.10	2.90	0.39
35–44	−0.13	1.11	−2.32	2.06	0.91
45–54	−1.32	1.13	−3.54	0.89	0.24
55–64	−2.14	1.23	−4.54	0.27	0.08
65–74	−2.04	1.75	−5.48	1.40	0.25
75+	−7.13	3.65	−14.29	0.03	0.05
**Gender**					
Female	3.17	0.51	2.18	4.16	0.00
Other	1.19	2.76	−4.23	6.62	0.67
**Education**					
Some college/no degree	3.25	1.65	0.02	6.48	0.05
Two-year	3.18	2.02	−0.79	7.16	0.12
Four-year	3.28	1.58	0.18	6.38	0.04
Master’s	2.11	1.60	−1.03	5.26	0.18
Doctoral	2.74	1.62	−0.43	5.92	0.09
Professional Degree (JD/MD)	1.47	1.76	−1.98	4.92	0.40
**Employment Status**					
Employed part-time	−0.25	0.86	−1.93	1.43	0.77
Unemployed	0.31	2.02	−3.65	4.27	0.87
Laid off due to COVID	1.97	2.09	−2.13	6.08	0.35
Retired	−0.95	2.78	−6.41	4.51	0.73
Student	1.82	1.00	−0.15	3.79	0.07
Other	−0.91	1.42	−3.69	1.87	0.52
**Income Bracket**					
10,000 to 50,000	1.30	1.06	−0.78	3.39	0.22
50,000 to 75,000	0.18	1.18	−2.12	2.49	0.87
75,000 to 100,000	0.15	1.25	−2.31	2.61	0.91
100,000 to 125,000	−0.47	1.24	−2.90	1.96	0.71
125,000 to 150,000	−0.15	1.25	−2.60	2.31	0.91
150,000 to 175,000	1.74	1.36	−0.92	4.40	0.20
175,000 to 200,000	1.11	1.32	−1.48	3.71	0.40
Greater than 200,000	0.45	1.12	−1.75	2.65	0.69
**Composites**					
Personal Burden Composite	0.66	0.41	−0.15	1.47	0.11
Personal Care Composite	−0.39	0.06	−0.50	−0.28	0.00
Family or Friends with COVID	0.60	0.55	−0.48	1.68	0.28
Hours of Daily Remote Work	−0.08	0.33	−0.73	0.57	0.80
Change in Work Expectations	−0.40	0.14	−0.67	−0.12	0.01
Increase in personal care Composite	−1.32	0.52	−2.35	−0.30	0.01
**U.S. Regions**					
Northeast	0.45	0.97	−1.46	2.36	0.64
South	−0.92	0.87	−2.62	0.79	0..29
Midwest	−0.15	1.16	−2.42	2.12	0.89
West	−1.36	0.72	−2.76	0.05	0.06
**Country (*n* > 30)**					
Pakistan	−2.43	1.09	−4.58	−0.28	0.03
Germany	−4.63	1.55	−7.68	−1.58	0.00
Canada	0.75	1.17	−1.54	3.04	0.52
India	0.08	2.02	−3.89	4.04	0.97
Netherlands	−1.77	1.38	−4.48	0.94	0.20
Mexico	−2.75	1.42	−5.48	−0.03	0.05
United Kingdom	−1.53	1.99	−4.33	1.27	0.28
China	−1.68	1.47	−5.59	2.23	0.40
Australia	−0.02	2.06	−2.93	2.88	0.98
Argentina	2.35	2.86	−1.69	6.41	0.25

## Supplementary Materials

The following are available online at https://www.mdpi.com/1660-4601/17/24/9248/s1, Supplementary Materials with Survey and Tables.
