# Supplementary material for: International Prevalence and Correlates of Psychological Stress during the Global COVID-19 Pandemic
Source: Int J Environ Res Public Health. 2020 Dec 10;17(24):9248. doi: 10.3390/ijerph17249248 (PMC7763004; doi:10.3390/ijerph17249248)
Supplement: Supplementary file 1 [file ijerph-17-09248-s001.pdf]

Supplementary Materials

For: Adamson MM, Phillips AL, et al., International prevalence and correlates of psychological stress during the global COVID-19 pandemic

Table of Contents

**Supplementary Materials**	1
**Tables**	
Table S1	12
Table S2	13
Table S3	14
Table S4	15

English

Block 1

Psychological Stress Associated with the COVID-19 Crisis

CONTACT: Dr. Maheen Adamson at 650-493-5000 ext 62179. To learn more about this Stanford University Study, please visit abslab.stanford.edu.

DESCRIPTION: You are invited to participate in a research study on psychological stress during the COVID-19 crisis. Our purpose is to measure the level of stress during this time and characterize it according to location, gender, income and other factors. This information will be collected through an online confidential survey. You will be asked to answer confidential survey questions that measure your level of stress during this time including questions about homeschooling, dependent care, full-time remote work, and social-distancing.

TIME INVOLVEMENT: Your participation will take approximately 8-10 mins.

RISKS AND BENEFITS: The risks associated with this study are minimal as it is an confidential survey. However, some questions related to stress may cause anxiety. We cannot and do not guarantee or promise that you will receive any benefits from this study.

PAYMENTS: You will receive no payment for your participation.

PARTICIPANT'S RIGHTS: If you have read this form and have decided to participate in this project, please understand your participation is voluntary and you have the right to withdraw your consent or discontinue participation at any time without penalty or loss of benefits to which you are otherwise entitled. The alternative is not to participate. You have the right to refuse to answer particular questions. The results of this research study may be presented at scientific or professional meetings or published in scientific journals. Your individual privacy will be maintained in all published and written data resulting from the study.

Are you an individual currently residing in the European Union (EU) / European Economic Area (EEA)?

Yes

No

General Data Protection Regulation Rights

For individuals in the EU/EEA: As described elsewhere in this Research Information Sheet, during the study, data pertaining to your participation in the study will be generated and recorded. In addition, we will collect from you only confidential data. We refer to all such data as “Your Study Data,” which will be specifically regulated in the EU/EEA under the General Data Protection Regulation (the “GDPR”). Your Study Data may be processed or used for the following purposes, which we refer to, collectively, as “Data Processing”:

- to carry out the study;
- to confirm the accuracy of the study;
- to monitor that the study complies with applicable laws as well as best practices developed by the research community;
- to make required reports to domestic and foreign regulatory agencies and government officials who have a duty to monitor and oversee studies like this one; and,
- to comply with legal and regulatory requirements, including requirements that data from this study, without information that could directly identify you, be made available to other researchers not affiliated with the study sponsor or with the study team. It is possible, for example, that as part of efforts to make research data more widely available to researchers, regulatory authorities in some countries may require that Your Study Data, without information that could directly identify you, be made publicly available on the internet or in other ways.

The following entities and organizations may engage in Data Processing of this study Data:

o the study team, including Dr. Maheen M. Adamson and her team.
o the study sponsor: Stanford School of Medicine
o Stanford Institutional Review Board

We may conduct the study in the United States or in other countries where the laws do not protect your privacy to the same extent as the laws in your country of residence. In addition, we may disclose confidential survey data for Data Processing to entities and individuals located in the United States or in other countries where the laws do not protect your privacy to the same extent as the laws in your country of residence. However, all reasonable steps will be taken to protect your privacy in accordance with the applicable data protection laws.

We have entered into a data transfer agreement with Stanford Medical School which is based on standard contractual clauses approved by the European Commission and ensures an adequate protection for Your Study Data. You may obtain a copy of the standard EU contractual terms by contacting the Principal Investigator, Maheen Mausoof Adamson, PhD at (650) 493-5000 ext 62179.

The GDPR gives you certain rights with regard to Your Study Data. You have the right to request access to, or rectification or erasure of, Your Study Data. You also have the right to object to or restrict our Data Processing of Your Study Data. Finally, you have a right to request that we move, copy or transfer Your Study Data to another organization. In order to make any such requests, please contact Principal Investigator, Maheen Mausoof Adamson, PhD at (650) 493-5000 ext 62179.

There is no limit on the length of time we will keep Your Study Data for this research because it may be analyzed for many years. We will also retain your Study Data to comply with our legal and regulatory requirements. We will keep it as long as it is useful, unless you decide you no longer want to take part. You are allowing access to this information indefinitely as long as you do not withdraw your consent.

You may withdraw your consent at any time. If you withdraw your consent, this will not affect the lawfulness or our collecting, use and sharing of Your Study Data up to the point in time that you withdraw your consent. Even if you withdraw your consent, we may still use Your Study Data that has been anonymized so that the data no longer identifies you. In addition, we may use and share Your Study Data that is anonymous as permitted by applicable law for purposes of: (a) public health (e.g., ensuring high standards quality and safety of health care and/or of medicinal products or medical devices), (b) scientific or historical research or statistical analysis as permitted by applicable European Union or European Union Member State laws and (c) archiving in the public interest.

CONTACT INFORMATION:
Questions: If you have any questions, concerns or complaints about this research, its procedures, risks and benefits, contact the Protocol Director, Maheen Mausoof Adamson, PhD, (650) 493 -5000 ext 62179. This survey is conducted in collaboration with the Stanford Department of Neurosurgery and the Stanford Department of Psychiatry. IRB Protocol #55809.

Independent Contact: If you are not satisfied with how this study is being conducted, or if you have any concerns, complaints, or general questions about the research or your rights as a participant, please contact the Stanford Institutional Review Board (IRB) to speak to someone independent of the research team at (650)-723-2480, or toll free at 1-866-680-2906. You can also write to the Stanford IRB, Stanford University, 1705 El Camino Real, Palo Alto, CA 94306.

Please print a copy of this page for your records. If you agree to participate in this research, please complete the following survey by clicking the right arrow.

Default Question Block

What is your marital status?

Married

Widowed

Divorced

Separated

Partnered

Single

Other

What is your age?

18-24 years old

25-34 years old

35-44 years old

45-54 years old

55-64 years old

65-74 years old

75 years or older

What is your level of education?

Less than high school degree

High school graduate (high school diploma or equivalent including GED)

Some college but no degree

Associate degree in college (2-year)

Bachelor's degree in college (4-year)

Master's degree

Doctoral degree

Professional degree (JD, MD)

Are you of Hispanic, Latino, or of Spanish origin?

Yes

No

Choose one or more races that you consider yourself to be.

White

Black or African American

American Indian or Alaska Native

Asian

Native Hawaiian or Pacific Islander

Other

Which statement best describes your current employment status?

Working (full-time)

Working (part-time)

Unemployed

Laid off or looking for work due to COVID-19

Retired

Not working due to disability

Student

Other

Which of the following industries most closely matches the one in which you are employed?

What is your gender?

Male

Female

Other

In which country do you currently reside?

Afghanistan
Albania
Algeria
Andorra
Angola
Antigua and Barbuda
Argentina
Armenia
Australia
Austria

In which state do you currently reside?

Alabama
Alaska
Arizona
Arkansas
California
Colorado
Connecticut
Delaware
District of Columbia
Florida

Please select your country's currency.

What is your annual household income in your selected currency?

Less than 10,000

10,000 to 50,000

50,000 to 75,000

75,000 to 100,000

100,00 to 125,000

125,000 to 150,000

150,000 to 175,000

175,000 to 200,000

Greater than 200,000

How many dependents do you have?

You are halfway through. Please make sure to click all the way through so that your responses are submitted.

Have any of your friends or family tested positive for COVID-19?

Yes

No

Are you providing homeschooling due to COVID-19?

Yes

No

N/A

Are you providing homecare (caretaking of elderly, disabilities, etc.) due to COVID-19?

Yes

No

N/A

Are you working remotely as of recent due to the COVID-19?

Yes

No

N/A

If so, how many hours a day?

< 1 hour

> 1 hour to 5 hours

> 5 hours to 8 hours

> 8 hours

How have the expectations of your work changed since COVID-19?

Much more

Moderately more

Slightly more

About the same

Slightly less

Moderately less

Much less

How much sleep are you getting currently during COVID-19?

0 - 2 hours

2 - 4 hours

4 - 6 hours

6 - 8 hours

8 - 10 hours

10 - 12 hours

Is this more or less than before COVID-19?

More

Less

Unchanged

How many minutes / hours a day are you currently getting regular exercise during COVID-19?

0

1-30 min

30min - 1hr

>1hr

Is this more or less than before COVID-19?

More

Less

Unchanged

How many minutes / hours a day are you currently using meditation during COVID-19?

0

1-30 min

30min - 1hr

>1hr

Is this more or less than before COVID-19?

More

Less

Unchanged

How many minutes / hours a day are you currently connecting with family / friends through telecommunication or virtually during COVID-19?

0

1-30 min

30min - 1hr

>1hr

Is this more or less than before COVID-19?

More

Less

Unchanged

Block 2

You are now beginning the Perceived Stress Scale (due to COVID-19). Please answer these questions as quickly and accurately as possible.

In the last month, how often have you been upset because of something that happened unexpectedly?

Never

Almost Never

Sometimes

Fairly Often

Very Often

In the last month, how often have you felt that you were unable to control the important things in your life?

Never

Almost Never

Sometimes

Fairly Often

Very Often

In the last month, how often have you felt nervous and stressed?

Never

Almost Never

Sometimes

Fairly Often

Very Often

In the last month, how often have you felt confident about your ability to handle your personal problems?

Never

Almost Never

Sometimes

Fairly Often

Very Often

In the last month, how often have you felt that things were going your way?

Never

Almost Never

Sometimes

Fairly Often

Very Often

In the last month, how often have you found that you could not cope with all the things that you had to do?

Never

Almost Never

Sometimes

Fairly Often

Very Often

In the last month, how often have you been able to control irritations in your life?

Never

Almost Never

Sometimes

Fairly Often

Very Often

In the last month, how often have you felt that you were on top of things?

Never

Almost Never

Sometimes

Fairly Often

Very Often

In the last month, how often have you been angered because of things that happened that were outside of your control?

Never

Almost Never

Sometimes

Fairly Often

Very Often

In the last month, how often have you felt difficulties were piling up so high that you could not overcome them?

Never

Almost Never

Sometimes

Fairly Often

Very Often

Block 3

Thank you again for your participation in this critical research. If you are concerned that you or a family member may be infected with COVID-19 (novel coronavirus), please contact your primary care physician or local healthcare provider. Additionally, please refer to CDC's and/or the WHO's guidelines and resources.
If you reside in the U.S., the National Suicide Prevention Lifeline provides free and confidential support that is available 24/7; you can call 1-800-273-8255 or chat online at https:/suicidepreventionlifeline.org/
If you would like to learn more about our research studies, please visit our website: abslab.stanford.edu.

Copyright © 2020 Stanford Medicine.

Table S1: Dated Survey Revisions

	Start date	End Date	Change 1	Change 2
**English**	4/8/2020	5/31/20	4/9/20	4/17/20
**Spanish**	4/13/2020	5/31/20	4/25/20	5/4/20
**Italian**	4/16/2020	5/31/20	..	..
**Traditional Chinese**	4/18/2020	5/31/20	..	..
**Simplified Chinese**	4/18/2020	5/31/20	..	..
**Japanese**	4/21/2020	5/31/20	..	..
**French**	4/22/2020	5/31/20	..	..

Table S2: Global Demographics

	F-Test	p-value
**Gender**	16·44	<·001
**Age**	5·87	<·001
**Education**	0·42	0·89
**Marital Status**	0·48	0·82
**Employment Status**	0·31	0·93
**Income**	1·05	0·37
**Industry**	1·23	0·22
**Number of dependents**	0·93	0·49

Table S3: U.S. Demographics

	F-Test	p-Value
**Gender**	12·87	<·001
**Age**	5·39	<·001
**Education**	0·91	0·49
**Marital Status**	0·79	0·58
**Employment Status**	1·04	0·39
**Income**	0·59	0·56
**Industry**	1·49	0·08
**Number of dependents**	0·29	0·94

Table S4: Survey Changes

	**Changes**
**4/9/2020**	Added Hispanic Question in addition to the “other” text box in race question, added marital status option of "single" rather than “partnered” in addition to "other" text box*
**4/15/2020**	Added between demographic questions and personal care and burden questions the line: "You are halfway through. Please make sure to click all the way through so that your responses are submitted."
**4/17/2020**	Clarified visibility for ends of questions on PSS-10 questions for mobile users
**4/17/2020**	In PSS-10, added "due to COVID-19" in prefacing text; added text response box in question 6 employment options; added "as of recent" after "working remotely" in Q17
**4/25/2020**	Translated "not employed" to "estudiante" in Spanish version*
**5/4/2020**	Translated introduction for EU users to Spanish

*Note. Survey respondents were able to respond in “other” categories, if needed.
